# Identification of claudin-2 as a promising biomarker for early diagnosis of pre-diabetes

**DOI:** 10.3389/fphar.2024.1370708

**Published:** 2024-02-15

**Authors:** Yang Songtao, Li Fangyu, Cao Jie, Yuan Li

**Affiliations:** Department of Endocrinology, Union Hospital, Tongji Medical College, Huazhong University of Science and Technology, Wuhan, China

**Keywords:** claudin-2, diabetes, impaired glucose tolerance, biomarker, pre diabetes

## Abstract

**Introduction:** Pre-diabetes, a high-risk metabolic state, is situated between normal glucose homeostasis and diabetes. Early identification of pre-diabetes offers opportunities for intervention and diabetes reversal, highlighting the crucial need to investigate reliable biomarkers for this condition.

**Methods:** We conducted an in-depth bioinformatics analysis of clinical samples from non-diabetic (ND), impaired glucose tolerance (IGT), and type 2 diabetes mellitus (T2DM) categories within the GSE164416 dataset. Thereafter the HFD and STZ treated mice were used for validation.

**Results:** This analysis identified several codifferentially expressed genes (Co-DEGs) for IGT and T2DM, including *CFB*, *TSHR*, *VNN2*, *APOC1*, *CLDN2*, *SLPI*, *LCN2*, *CXCL17*, *FAIM2*, and *REG3A*. Validation of these genes and the determination of ROC curves were performed using the GSE76895 dataset. Thereafter, *CLDN2* was selected for further verification. Gene expression analysis and immunofluorescence analysis revealed a significant upregulation of *CLDN2* expression in the pancreas islets of mice in the high-fat diet and T2DM groups compared to the control group. Similarly, serum level of *CLDN2* in patients with IGT and T2DM were significantly higher than those in the healthy group.

**Discussion:** These results suggest that *CLDN2* can serve as a novel biomarker for pre-diabetes, providing a new direction for future research in the prevention of type 2 diabetes.

## 1 Introduction

The incidence of metabolic syndrome and diabetes has been steadily increasing over the years, imposing significant economic burdens, with a notable trend of affecting younger individuals (Rooney et al., 2023). Which causing the prevalence of pre-diabetes has reached alarming levels, thereby affecting a substantial portion of the global population. Pre-diabetes is a condition where the blood glucose levels are higher than normal but not yet high enough for a diabetes diagnosis. This phase led to an increased risk of developing type 2 diabetes and cardiovascular diseases. Pre-diabetes carries a lifetime risk of conversion to T2DM, reaching up to 70%, and is linked to increased risks of cardiovascular disease and all-cause mortality (Cai et al., 2021). Early identification and targeted interventions during the pre-diabetic phase can significantly reduce the risk of diabetes and its associated comorbidities. However, this condition often goes undetected as individuals may not experience noticeable symptoms. While inflammatory factors, metabolic byproducts, non-coding RNAs have laid the foundation for novel pathophysiological hypotheses and treatments for pre-diabetes, no biomarkers have yet been incorporated into routine screening. In this backdrop, the quest for early biomarkers that can reliably signify the onset of pre-diabetes has gained prominence in biomedical research.

By collecting and analyzing GEO data from normal, pre-diabetic, and diabetic patients, we screened out many different genes. Amidst the myriad candidates under scrutiny, one gene that has emerged as a potential biomarker is Claudin-2 (*CLDN2*). *CLDN2* is a member of tight junctions transmembrane proteins family (Ganapathy et al., 2022). Normally, *CLDN2* mRNA is enriched in the kidney, gastrointestinal tract, liver, gall bladder, and pancreas (Venugopal et al., 2019). And it is mainly reported the permeability function of *CLDN2* in intestinal diseases and cancer (Zeisel et al., 2019). CLDN2 plays an important role in inflammatory diseases. It has reported that CLDN2 expression was elevated in active inflammatory bowel disease (IBD), adenomas, and IBD-associated dysplasia (Weber et al., 2008). In addition, Claudin-2 protein abundance is thought to correlate with pancreatic tissue inflammation (Whitcomb et al., 2012), supporting a role in pancreatic inflammation. Recent findings have expanded our understanding of *CLDN2*, revealing its role in signal modulation, including the regulation of cell proliferation, migration, signaling pathways, and transcription factors (Venugopal et al., 2019). Besides, the role of *CLDN2* in T1DM nephropathy and its impact on the pancreas have further strengthened the association between *CLDN2* and diabetes (Molina-Jijon et al., 2014; Aghdassi et al., 2015). However, the exact relationship between *CLDN2* and pre-diabetes is unclear.

The early and effective identification of pre-diabetic conditions is crucial for preventing the progression from pre-diabetes to diabetes and potentially reversing the pre-diabetic state. Here, We identified that *CLDN2* is a potential biomarkers for early diagnosis of pre-diabetes through bioinformatics. Further validation revealed that *CLDN2* is upregulated both in mice fed a high-fat diet (HFD) with or without STZ treatment, while with a higher ratio in STZ mice. The serum of volunteers in impaired glucose tolerance (IGT) group and diabetes mellitus (DM) group also showed higher *CLDN2* level. In this study, we attempt to identify the promising biomarkers for the recognition of pre-diabetic states at an early stage. Which serves as a critical stone in the broader context of diabetes prevention and management. Efforts directed towards early identification and intervention strategies are pivotal for mitigating the escalating global burden of diabetes. Our findings have offered valuable assistance in delving deeper into the research on pre-diabetes biomarkers, presenting new insights into the study of pre-diabetes molecular complexity.

## 2 Materials and methods

### 2.1 Study population

The clinical samples involved in the experiment were all from our previous studies (Yang et al., 2023) and the study was conducted according to the guidelines of the Declaration of Helsinki, and approved by The Ethics Committee of Tongji Medical College, Huazhong University of Science and Technology (IORG No: IORG0003571).

### 2.2 Data collection

The expression profiling of GSE164416, which is based on the GPL16791 platform [Illumina HiSeq 2,500 (*Homo sapiens*), United States], was downloaded from the GEO database. The dataset contains pancreatic tissue samples from 15 non-diabetic (ND) patients, 13 patients with T2DM, and 18 patients with impaired glucose tolerance (IGT). Only patients with the age between 30–70 years old and BMI between 20–28 kg/m^2^ were recruited.

### 2.3 Data preprocessing

DEGs were screened between the ND and IGT groups and between the ND and T2DM groups, respectively, using the “limma” package in the R software, with parameters set to adj.*p*-value <0.05 and |log2FC| ≥ 0. Next, we plotted volcano maps and heat maps using the “ggplot2” and “heat map” packages.

### 2.4 Enrichment analysis

Gene Ontology (GO) enrichment analysis was carried out on the webpage using the Database for Annotation, Visualization, and Integrated Discovery (DAVID; https://davi.ncifcrf.gov) online tools, including molecular functions (MFs), cellular components (CCs), and biological processes (BPs). Kyoto Encyclopedia of Genes and Genomes (KEGG) pathways for functional annotation and pathway enrichment analysis of the DEGs.

### 2.5 Validation of hub genes

Pancreatic tissue gene expression profiles from the GSE76895 dataset were used to validate hub genes. The microarray data were based on the GPL570 (HG-U133_Plus_2) Affymetrix Human Genome U133 Plus 2.0 array (Affymetrix, Santa Clara, CA, United States). Twenty-five patients with T2DM and 25 patients with ND were selected from the GSE76895 dataset, and the differences in the expression of hub genes in GSE76895 were plotted as box plots using the draw_boxplot function of the online tool BioLadder (https://www.bioladder.cn/). ROC curves for the selected genes were also calculated using BioLadder to assess the ability of the hub genes to diagnose T2DM.

### 2.6 Animal model

6-week-old male C57BL/6J mice were obtained from Beijing HFK Biotechnology Co., Ltd. The standard-diet (SD) group was fed a standard chow diet (SD, 20% protein, 70% carbohydrates, and 10% fat). The high-fat diet (HFD) group was fed a high-fat diet (HFD; Research Diets, D12492, 20% protein, 20% carbohydrates, and 60% fat) for 3 months to induce insulin resistance. The streptozotocin (STZ) group was fed a HFD and treated with STZ (30 mg/kg intraperitoneally injection) to establish STZ-induced diabetic mouse. The mice in the SD and the HFD group were intraperitoneally injected with PBS. The mice were sacrificed at the week 16. All mice were housed in a specific pathogen-free animal laboratory in a 12-h light and 12-h darkness cycle at room temperature. The body weight, food intake, and fasting blood glucose (FBG) of mice were measured every 2 weeks. All of the experiments were performed according to procedures approved by the Animal Research Committee of Tongji Medical College, Huazhong University of Science and Technology, Hubei Province, China. (IACUC Number: 3417).

### 2.7 FBG, IPITT and IPGTT

Mice fasting blood glucose (FBG) was measured using a blood glucose meter (LifeScan) after mice were fasted overnight. The intraperitoneal insulin tolerance tests (IPITT) were performed in mice by the injection of insulin (0.75 U/kg, i.p.) after mice were fasted overnight, and the intraperitoneal glucose tolerance tests (IPGTT) were performed by injecting glucose (2 g/kg, i.p.) after mice were fasted overnight; plasma glucose was measured at different time points.

### 2.8 Western blotting

Protein from cells or pancreatic tissue was extracted in RIPA lysis buffer (NCE Biotech, China) with protease and phosphatase inhibitors. Protein samples were separated by SDS-PAGE and transferred to a PVDF membrane (Millipore, United States). The membrane was incubated with primary antibodies (Rabbit Anti-*CLDN2*, diluted 1:1,000, Proteintech, 26912-1-AP, China; Mouse anti-β-actin, diluted 1:2000, 66009-1-lg, Proteintech, China) overnight at 4°C and were incubated in secondary antibodies (diluted 1:3,000) for 1 h at room temperature. Finally, the membranes were visualized with an ECL reagent (NCE Biotech, China) and measured. The Protein Marker was 180 kDa Plus Prestained Protein Marker (MP102, Vazyme, China).

### 2.9 qRT-PCR

Total RNA from pancreas tissue was extracted using TRIzol reagent (Takara Shuzo Co., Ltd., Kyoto, Japan), and the cDNA was synthesized using a Prime Script RT reagent kit (Takara Biotechnology Co. Ltd., Japan). Then qPCR was performed in a LightCycler480-PCR system (Roche Diagnostics, Mannheim). The relative transcript levels of the objective gene were normalized to β-actin and calculated by the 2^−ΔΔCT^ method. The primer sequences are listed as follows: *CLDN2*- Forward sequence (5′–3′) cat​cac​cca​gtg​cga​tat​cta​ca, *CLDN2*- Reverse sequence (5′–3′) gag​ata​ata​caa​gcc​agc​gag​ga. β-actin-Forward sequence (5′–3′) att​gtt​acc​aac​tgg​gac​g, β-actin-Reverse sequence (5′–3′) ctg​ggt​cat​ctt​ttc​acg.

### 2.10 Immunofluorescence

The paraffin sections of pancreas were deparaffinized with xylene and then dehydrated in 100%, 95%, 85%, 70% ethanol for 5 min, respectively. The sections were incubated with primary antibodies overnight at 4°C (Rabbit Anti-CLDN2, diluted 1:100, Proteintech, 26912-1-AP, China; Mouse anti-insulin, diluted 1:100, 66198-1-Ig, proteintech; Rabbit anti-glucagon, diluted 1:100, Ab92517, Abcam). After PBS washing, the sections were incubated with secondary antibody (FITC, CY3, Servicebio) for 1 h and nuclei were stained with DAPI (Servicebio, Wuhan, China) at room in the dark. The images were analyzed by a confocal microscope (Nikon, Tokyo, Japan).

### 2.11 ELISA

The *CLDN2* levels were measured using a commercial ELISA kit (HM12058, Bioswamp Life Science Lab, Wuhan, China) following the manufacturer’s instructions. The IL1β and TNFα levels were measured using a commercial ELISA kit (KE10003, KE20018, Proteintech, Wuhan, China) following the manufacturer’s instructions. The absorbance at 450 nm was detected using a microplate reader (PerkinElmer, Waltham, MA, United States).

### 2.12 Statistical analysis

The data was analyzed and processed by GraphPad Prism 9.5 software. Differences in numeric parameters between two groups were assessed with an unpaired two-tailed *t*-test, and one-way or two-way ANOVA was used for comparison among multiple groups. All data were presented as the mean ± standard deviation, and *p* <0.05 was considered statistically significant. The entirety of the experiments was repeated at least three times.

## 3 Results

### 3.1 Identification of DEGs for IGT and T2DM

**FIGURE 1:**
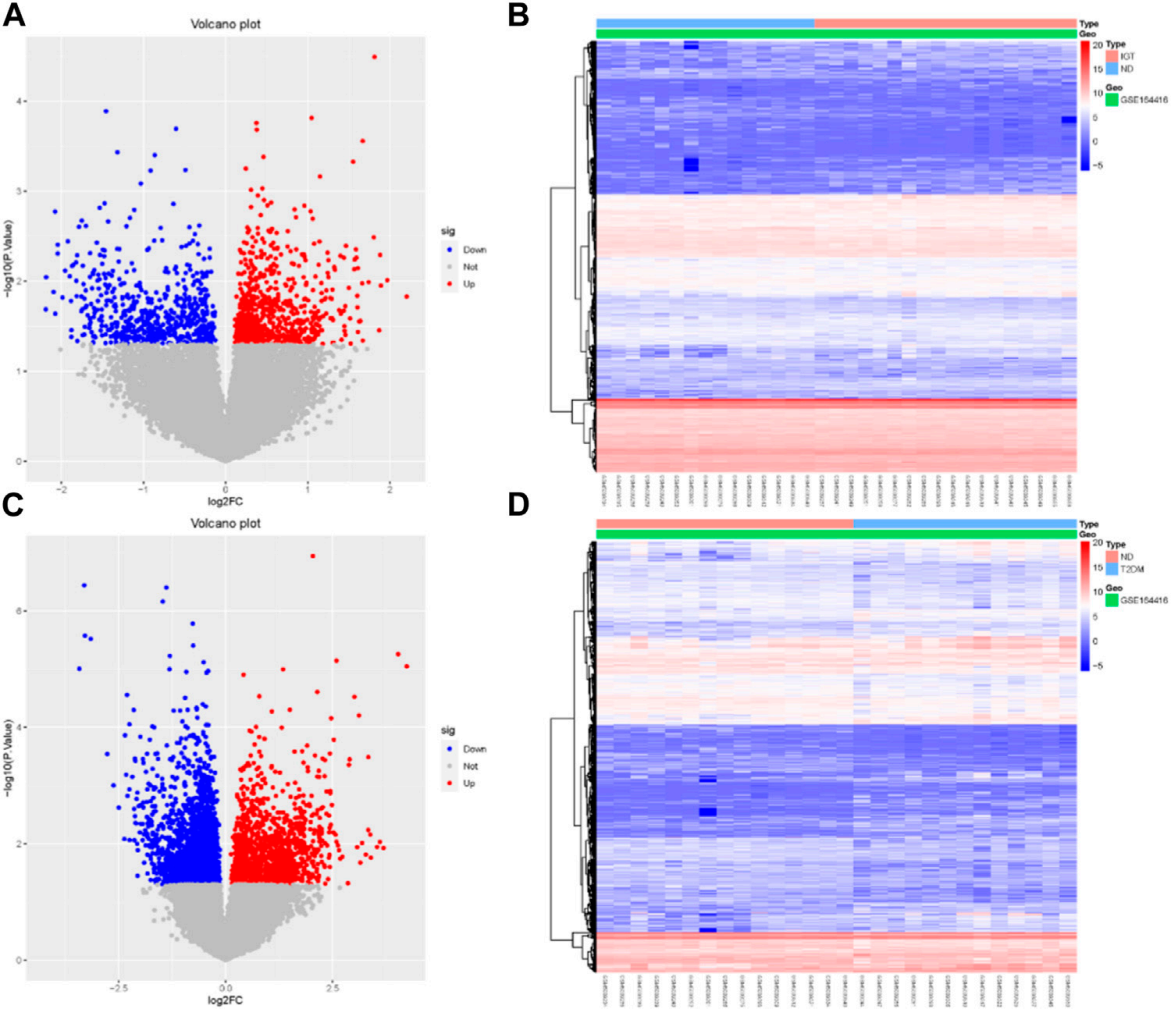
Volcano map **(A, C)** and heat map **(B, D)** of DEGs for IGT and T2DM.

Using the R package “limma,” we identified 737 upregulated genes and 595 downregulated genes in the GSE164416 dataset for IGT samples compared to ND samples. Volcano diagram, heat map of DEGs in IGT shown in Figures 1A, B.

The same method was used to analyze the DEGs in the T2DM samples, and we found that there were 3590 DEGs, including 1,399 upregulated genes and 2,191 downregulated genes. The results were mapped as a volcano map (Figure 1C) and a heat map (Figure 1D).

We select the differential genes from T2DM with |log2FC| ≥ 1.5 and take the intersection of the differential genes from IGT with |log2FC| ≥ 0 to obtain the Co-DEGs, namely, *CFB*, *TSHR*, *VNN2*, *APOC1*, *CLDN2*, *SLPI*, *LCN2*, *CXCL17*, *FAIM2*, *REG3A*.

### 3.2 GO and KEGG pathway analysis

**FIGURE 2:**
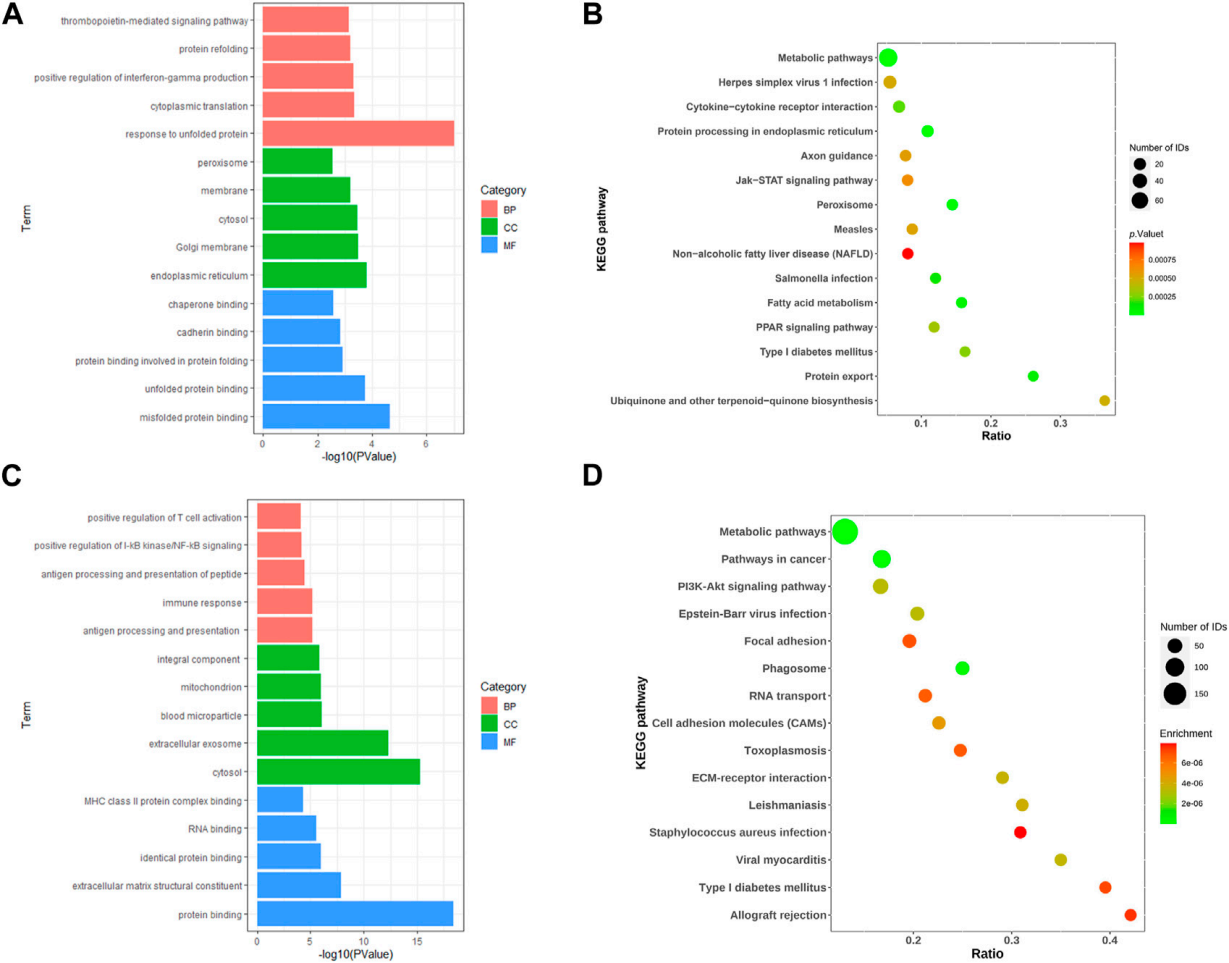
Go of DEGs **(A, C)** and KEGG of DEGs **(B, D)** for IGT and T2DM.

We performed enrichment analysis of the GO terms and KEGG pathways of differential genes for IGT. In the biological process (BP), the DEGs were mainly enriched in the thrombopoietin-mediated signaling pathway and the protein refolding and positive regulation of interferon-gamma production. As for the cellular component (CC) group, DEGs were mainly enriched in peroxisome, membrane and cytosol. In molecular function (MF), DEGs were mainly enriched in protein binding involved inchaperone binding, cadherin binding and protein folding (Figure 2A). KEGG pathway enrichment analysis suggested that the DEGs mainly played key roles in metabolic pathways, protein processing in endoplasmic reticulum, fatty acid metabolism (Figure 2B).

Similarly, GO enrichment analyses and KEGG pathway for T2DM DEGs. The BPs were mainly focused on positive regulation of T cell activation, positive regulation of I-κB kinase/NF-κB signaling and antigen processing and presentation of peptide; CCs were mainly focused on the integral component, mitochondrion and blood microparticle; and MFs were mainly focused on class II protein complex binding, RNA binding an MHC identical protein binding (Figure 2C). In addition, the KEGG pathway analysis showed that the DEGs were closely related to the metabolic pathways, pathways in cancer and PI3K-Akt signaling pathway (Figure 2D).

### 3.3 Validation of hub genes

**FIGURE 3:**
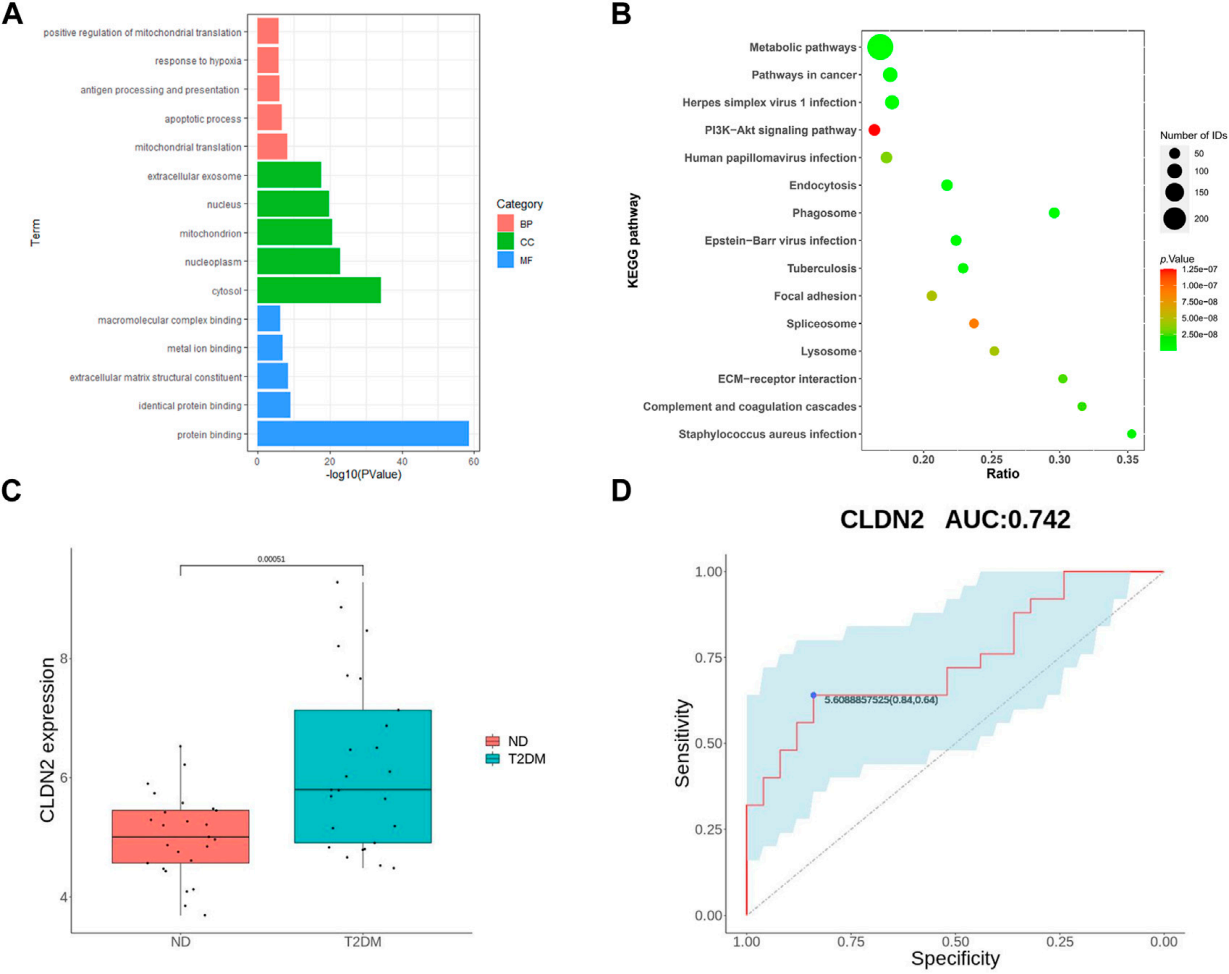
Validation of hub genes: GO of DEGs **(A)** and KEGG of DEGs **(B)** for T2DM; **(C)** *CLDN2* expression in GSE76895; and **(D)** ROC curves of the *CLDN2* gene in GSE76895.

To validate the screened genes, we analyzed the expression of the pivotal genes in the gene expression profiles of pancreatic tissues in the GSE76895 dataset (comprising 25 ND and 25 T2DM). We performed enrichment analyses of ND and T2DM differential genes in GES76895, and the results obtained were highly consistent with GSE164416, with the top 5 ranked overlaps including: antigen processing and presentation of exogenous peptide antigen via MHC class II in the BPs; cytosol, mitochondrion, extracellular exosome in the CCs; protein binding, identical protein binding, extracellular matrix structural constituent in the MFs (Figure 3A). Similarly, 8 of the top 15 KEGG pathways were consistent between GES76895 and GSE164416, including, Metabolic pathways, Phagosome, Pathways in cancer, Epstein-Barr virus infection, *Staphylococcus aureus* infection, ECM-receptor interaction, Focal adhesion, PI3K-Akt signaling pathway (Figure 3B). As shown in Figure 3C, *CLDN2* expression was significantly upregulated in pancreatic islets from T2DM patients compared to controls (*p* < 0.05). In addition, we plotted ROC curves for the central genes and calculated the area under the curve (AUC) to differentiate between the T2DM and controls. The diagnostic value AUC for the *CLDN2* gene in GSE76895 was 0.742 (Figure 3D).

### 3.4 The establish of mouse models of pre-diabetes and T2DM

**FIGURE 4:**
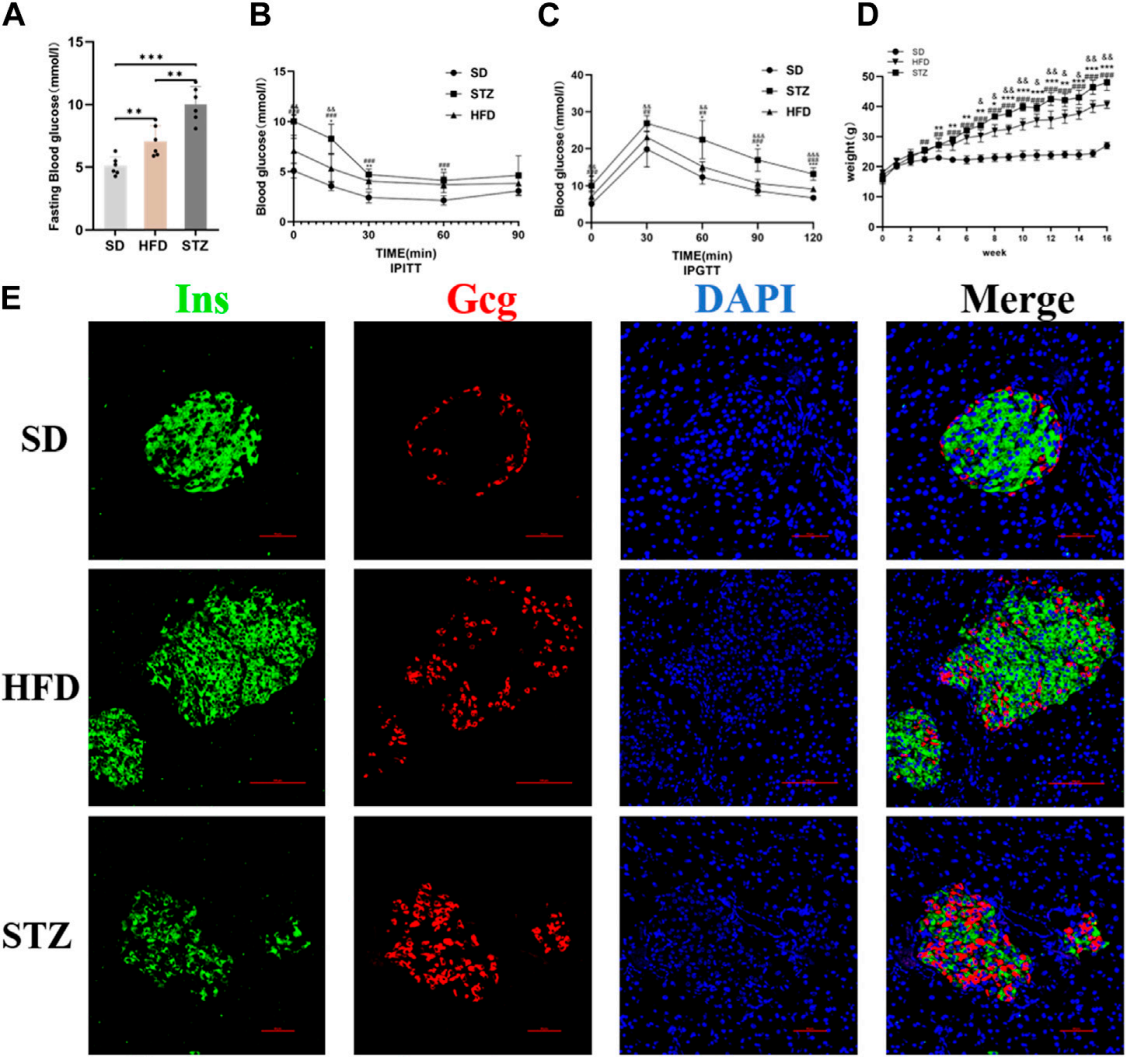
Validation of the Establishment of Mouse Models of Pre-diabetes and T2DM. Fasting blood glucose **(A)**, IPITT **(B)**, IPGTT **(C)** and Body weight **(D)** of mice in SD, HFD and STZ group. **(B, C)** *p < 0.05, **p < 0.01, ***p < 0.001 in SD vs. HFD; #*p* < 0.05, ##*p* < 0.01, ###*p* < 0.001 in SD vs. STZ; &*p* < 0.05, &&*p* < 0.01, &&&*p* < 0.001 in HFD vs. STZ. **(E)** Representative immunofluorescent images of insulin and glucagon in the pancreatic islets. Bar = 50 nm.

Following the bioinformatics analysis, we observed a significant upregulation of *CLDN2* expression in the pancreas of volunteers with IGT and T2DM. To further validate the alterations of *CLDN2* in pre-diabetes and T2DM, we initially established the metabolic stress model of mice to create pre-diabetic conditions. Also, STZ mice were used to assess the diabetic state. We observed that mice fed a HFD and STZ mice exhibited significantly higher body weights and fasting blood glucose levels compared to the SD group (Figures 4A, D). As anticipated, these mice displayed evident impairments in glucose tolerance and insulin resistance (Figures 4B, C). Thereafter, immunofluorescence results revealed an increase in pancreatic islets α-cell numbers and a substantial loss of β-cells in the HFD group, with a more pronounced effect observed in STZ mice, leading to a dysregulation in the α/β-cell ratio (Figure 4E). These findings indicate the successful establishment of mouse models for pre-diabetes and diabetes.

### 3.5 *CLDN2* is upregulated in pancreatic islets of pre-diabetes and T2DM mouse models

**FIGURE 5:**
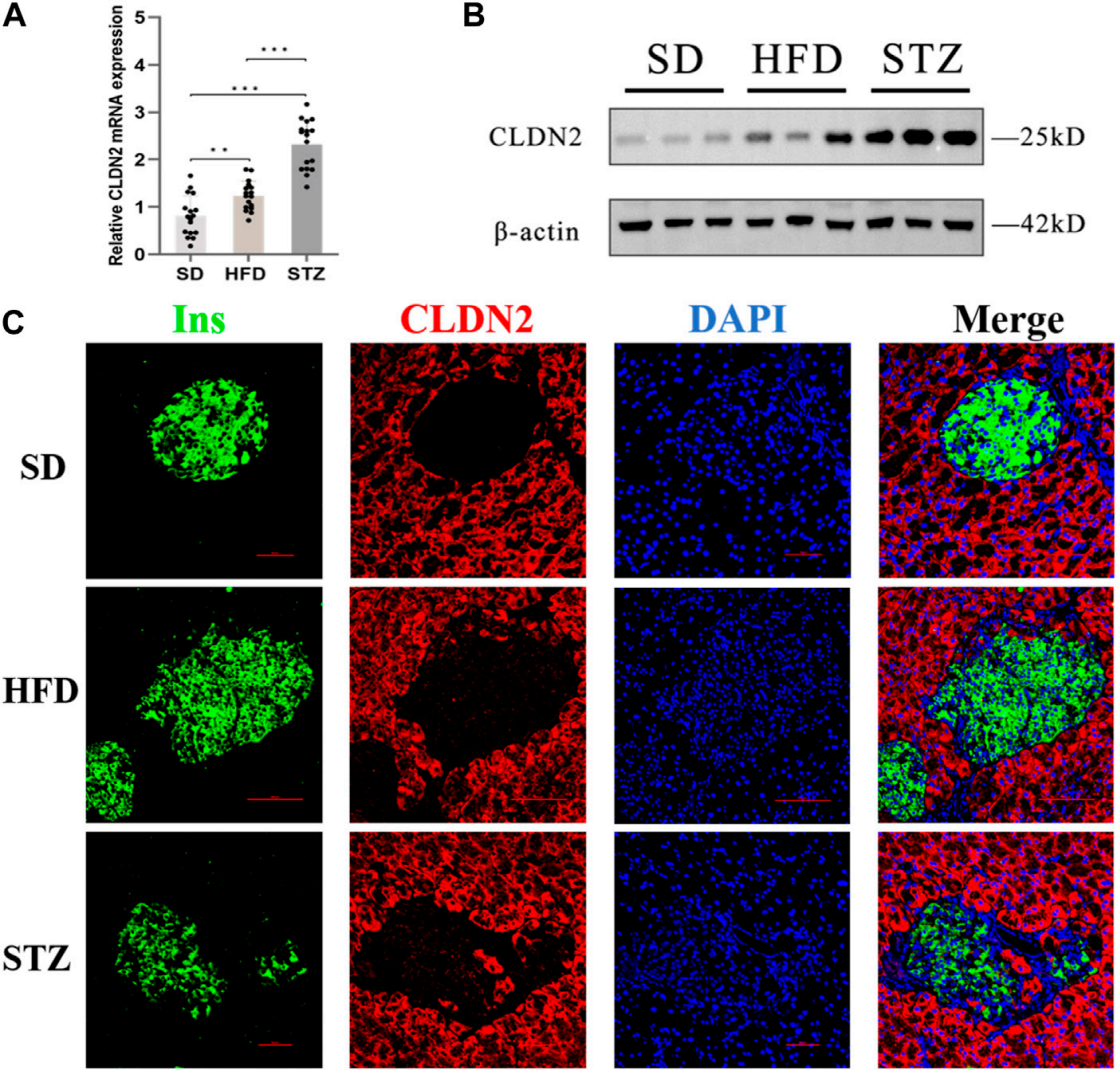
The expression level of *CLDN2* in pancreatic islets of pre-diabetes and T2DM mouse models. **(A)** *CLDN2* expression was determined using real-time quantitative RT-PCR (***p < 0.001) **(B)** Western blot showing the overexpression of *CLDN2* in both HFD and STZ mice. **(C)** Representative immunofluorescent images of insulin and *CLDN2* in the pancreatic islets. Bar = 50 nm.

To validate alterations of *CLDN2* in pre-diabetes and T2DM, we assessed the expression levels of *CLDN2* in the pancreas of mice. Transcriptional level verification indicated a significant increase in *CLDN2* mRNA in both the HFD and STZ mice, with a more remarkable increase observed in STZ mice (Figure 5A). Western blot showed the same results, revealing a substantial increase in *CLDN2* protein expression in both the HFD and STZ groups compared to the SD group, with a particularly notable elevation in the STZ group (Figure 5B). To investigate whether *CLDN2* is upregulated within the pancreatic islets, we conducted immunofluorescence staining of the islets. Confocal microscopy observations revealed that, under normal conditions, *CLDN2* is predominantly expressed in pancreatic tissue but exhibits almost expressionless within the islets. However, with changes in metabolic conditions, not only did *CLDN2* expression significantly increase in the peripheral regions of the islets, but expression within the islets themselves was also evident (Figure 5C). Notably, within the islets of STZ mice, *CLDN2* expression was significantly elevated, suggesting a compensatory increase possibly associated with excessive loss or fibrosis of islet cells (Figure 5C).

### 3.6 Verification of *CLDN2* to be the potential pre-diabetes biomarker in clinical samples

**FIGURE 6:**
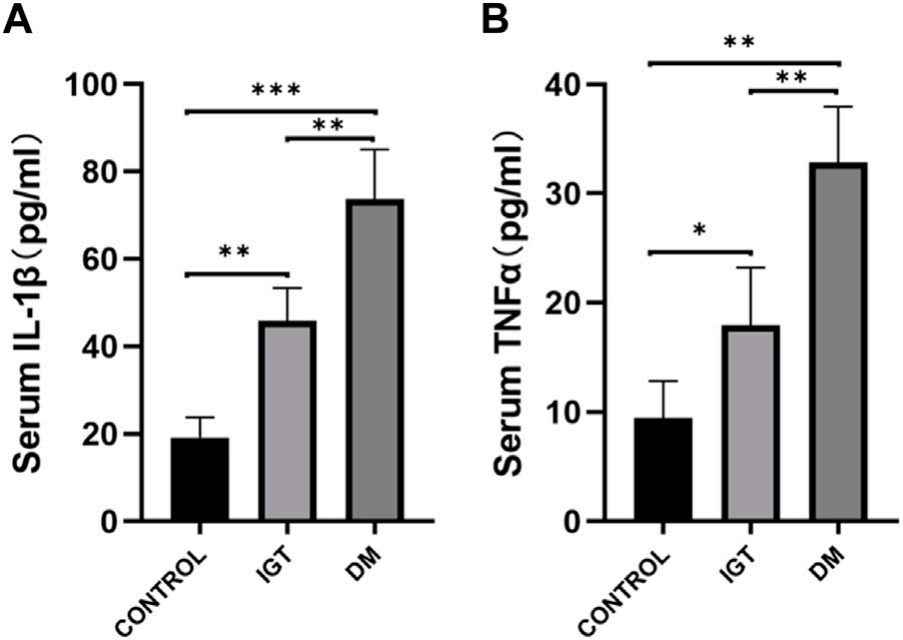
Comparison of serum IL1β **(A)** and TNFα **(B)** level among the IGT group, the T2DM group, and the control (*p < 0.05, **p < 0.01, ***p < 0.001).

To validate the clinical utility potential of the *CLDN2* gene, we used ELISA to assess the protein levels encoded by the *CLDN2* gene in clinical serum samples. Blood glucose and HbA1C level in both the IGT and T2DM groups were significantly higher than those in the control group, and these differences were statistically significant (Table 1). The serum expression levels of *CLDN2* in both the IGT and T2DM groups were markedly elevated compared to the control group, with statistically significant differences. These findings indicate that the expression of *CLDN2* have changed in the early stages of metabolic stress. The identified *CLDN2* gene holds promise as a potential biomarker for early diagnosis and prognosis prediction in pre-diabetes. Besides, the association between CLDN2 level and clinical parameters was investigated by Pearson correlation coefficient test, the results were shown in Table 2. We found that FBG in the IGT group was correlated with CLDN2 level. But HbA1C were not correlated with clinical features. We think this may be due to some patients consistently taking antidiabetic medication before admission, and since HbA1C represents a 3-month average, there might be a certain lag, resulting in a weak correlation between the two. Therefore, it is necessary to expand the sample size and include patients who have not taken any medication for further correlation experiments. Further, we assessed the expression levels of serum IL1b and TNFα (Figure 6). We observed a significant upregulation of these cytokines in populations from the Impaired IGT and DM groups. Previous studies have indicated that the elevation of IL1b and TNFα can upregulate the expression of CLDN2 (Yamamoto et al., 2004; Menta et al., 2022; Wu et al., 2022). Therefore, the upregulation of CLDN2 expression in our study may be regulated by the increased levels of these inflammatory factors.

**TABLE 1:** Comparison of data of volunteers in three groups.

	Control (*n* = 17)	IGT (*n* = 19)	DM (*n* = 24)
Age (year)	36	43*	57*
FBG (mmol/L)	4.7	6.9*	9.1**
HbA1C (%)	5.12	6.31**	8.74**
*CLDN2* (ng/mL)	2.15	3.29***	4.27***

(*p < 0.05, **p < 0.01, ***p < 0.001 vs. control).

**TABLE 2:** Correlation of CLDN2 level with clinical parameters.

	CLDN2			
	IGT		DM	
	r	*p*	r	*p*
FBG	0.474913	0.037195	0.244579	0.855349
HbA1C	0.188201	0.000211	−0.372706	0.577914

## 4 Discussion

The early identification and targeted intervention of pre-diabetes can significantly reduce the risk of diabetes and its associated complications, such as cardiovascular diseases and all-cause mortality. Therefore, the investigation for early biomarkers that can conveniently and reliably indicate the onset of pre-diabetes is crucial. In this study, we obtained the GSE164416 dataset from GEO and identified 737 upregulated genes and 595 downregulated genes in IGT, as well as 1,399 upregulated genes and 2,191 downregulated genes in T2DM through differential expression analysis. Further analysis led to the identification of 10 candidate genes, namely, *CFB*, *TSHR*, *VNN2*, *APOC1*, *CLDN2*, *SLPI*, *LCN2*, *CXCL17*, *FAIM2*, and *REG3A*. Subsequently, we validated the expression and diagnostic value of these key genes using the GSE76895 dataset. Finally, we determined that *CLDN2* is the most promising gene in terms of diagnostic potential in the GSE164416 and GSE76895 datasets.

Research on *CLDN2* has primarily focused on gastrointestinal diseases, due to its role as a member of the tight junction protein family, prompting extensive investigation into its role about the intestinal barrier (Beggs et al., 2023; Ganapathy et al., 2023; Rini et al., 2023). Numerous studies have progressively affirmed its association with inflammatory bowel diseases (Raju et al., 2020; Ahmad et al., 2023; Alghamdi and Al-Zahrani, 2023). Besides, researches on the association between *CLDN2* and cancer has been steadily increasing (Piontek et al., 2020), with a growing evidence elucidating its involvement in tumor growth, metastasis, and metabolism (Eguchi et al., 2021; Hirota et al., 2021; Tabaries et al., 2021; Wei et al., 2021). However, although several studies have explored the possible role of *CLDN2* in T1DM (Sapone et al., 2006; Visser et al., 2010; Molina-Jijon et al., 2014), the relationship between *CLDN2* and diabetes remained unclear. Recent studies have gradually revealed that *CLDN2*, distinct from its conventional permeability function, may also regulate biological activities and play a role in signal transduction, such as proliferation, migration and signal-modulating (Ikari et al., 2012; Zhang et al., 2018; Venugopal et al., 2019; Wang et al., 2019). Combined with our bioinformatics analysis, we considered that *CLDN2* has the potential of a pre-diabetes biomarker. We established mouse models for both pre-diabetes and diabetes, validating a significant upregulation of *CLDN2* expression in both disease models, with higher expression levels in the diabetes group. This indicates an intricate association between *CLDN2* and the onset and progression of diabetes, indicating that the *CLDN2* expression has altered during metabolic stress in the pre-diabetic period.

Another important aspect is evident in our immunofluorescence results, revealing that in physiological conditions, *CLDN2* primarily localizes to the peripheral tissue surrounding the pancreatic islets. This may reflect its role in barrier function or permeability. However, under metabolic stress, *CLDN2* not only exhibits a significant increase in expression around the islets but also appears within the islets. In the period of diabetes, there is a dramatic increase in *CLDN2* expression within the islets. This suggests that under normal conditions, *CLDN2* in the pancreas may primarily contribute to maintaining normal physiological function through its tight junction-related role. The excessive expression of *CLDN2* within the islets of STZ mice during diabetes state may function as a protective barrier to prevent further damage to islet cells or play a compensatory regulatory role, such as signaling pathways related to islet cell proliferation. However, the mechanisms underlying this phenomenon require further research.

To validate the clinical application potential of *CLDN2*, we conducted ELISA to measure serum *CLDN2* protein levels in volunteers with IGT and T2DM. Our results confirmed the potential of *CLDN2* as a pre-diabetes biomarker and indicated its potential involvement in the pathogenesis of diabetes. Therefore, we believe that assessing *CLDN2* levels may valuable in the diagnosis and assessment of pre-diabetes, which is also the clinical significance of this study. The primary limitations of this study are as follows: 1. The limited size of the clinical sample may introduce potential biases to the results. We intend to expand the sample size to mitigate this limitation in the future. 2. The role of *CLDN2* in IGT and diabetes requires further elucidation, particularly beyond its functions related to barrier and permeability. 3. Additional genes identified through bioinformatics analysis need further exploration to understand their potential connections with diabetes. In summary, this study introduces novel biomarkers for pre-diabetes diagnosis and opens new horizon for research into the prevention of type 2 diabetes.

## Data Availability

The raw data supporting the conclusion of this article will be made available by the authors, without undue reservation.
