# Supplementary figures and images for: Identification of claudin-2 as a promising biomarker for early diagnosis of pre-diabetes
Source: Front Pharmacol. 2024 Feb 15;15:1370708. doi: 10.3389/fphar.2024.1370708 (PMC10902111; doi:10.3389/fphar.2024.1370708)

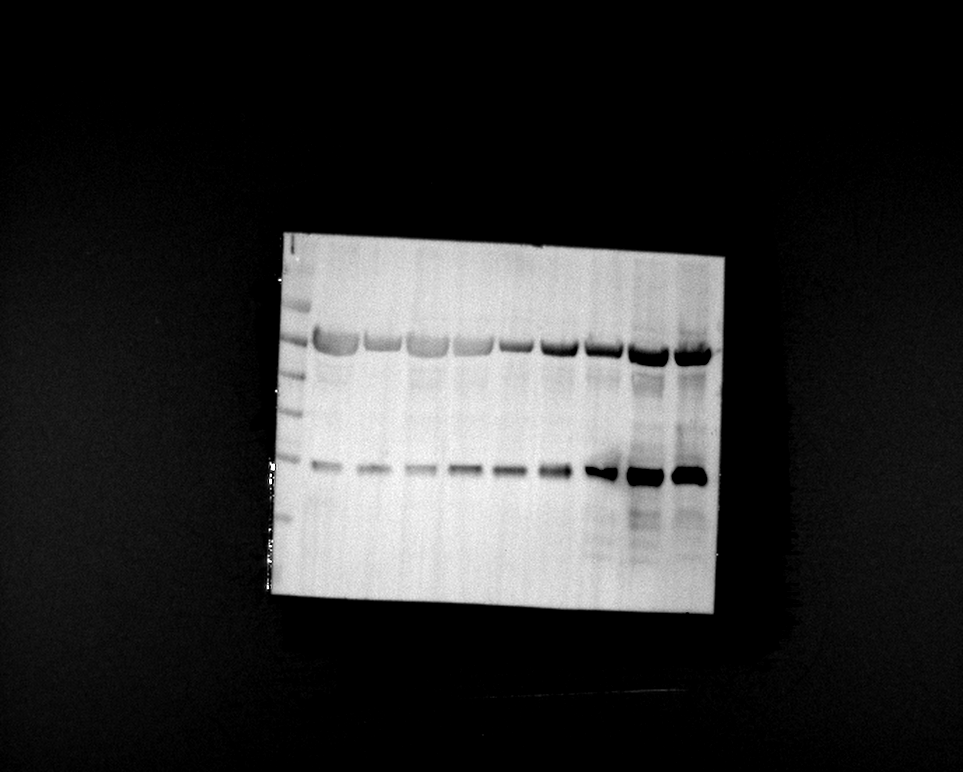

Supplement: Supplementary file 1 [file DataSheet1.ZIP › WB/1.tif]

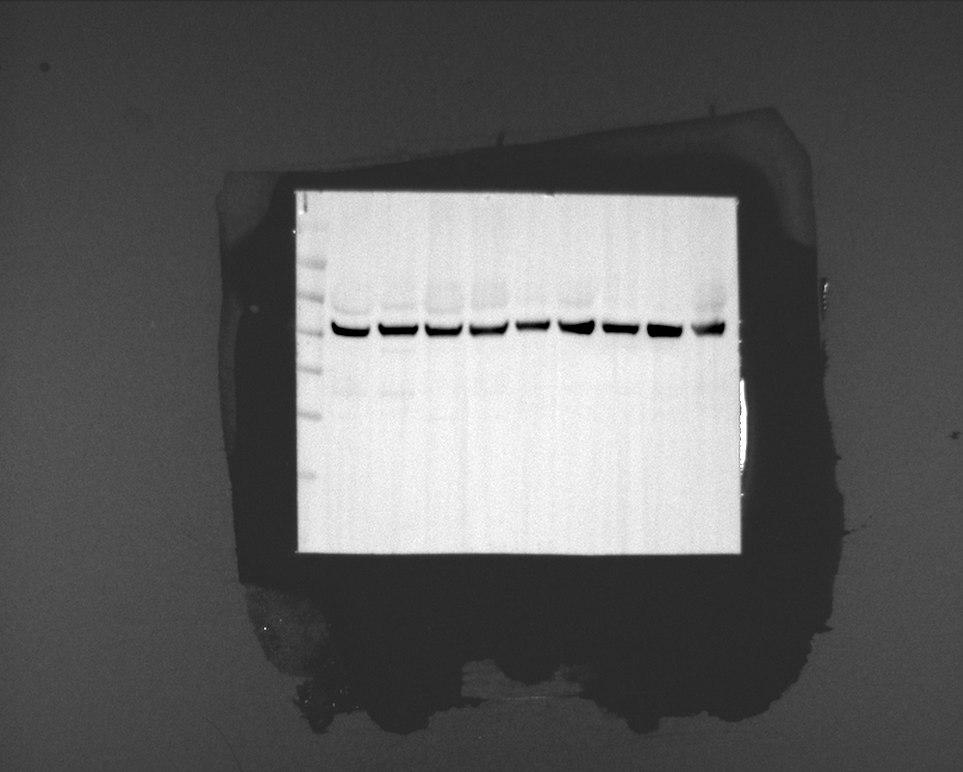

Supplement: Supplementary file 1 [file DataSheet1.ZIP › WB/1actin1.tif]

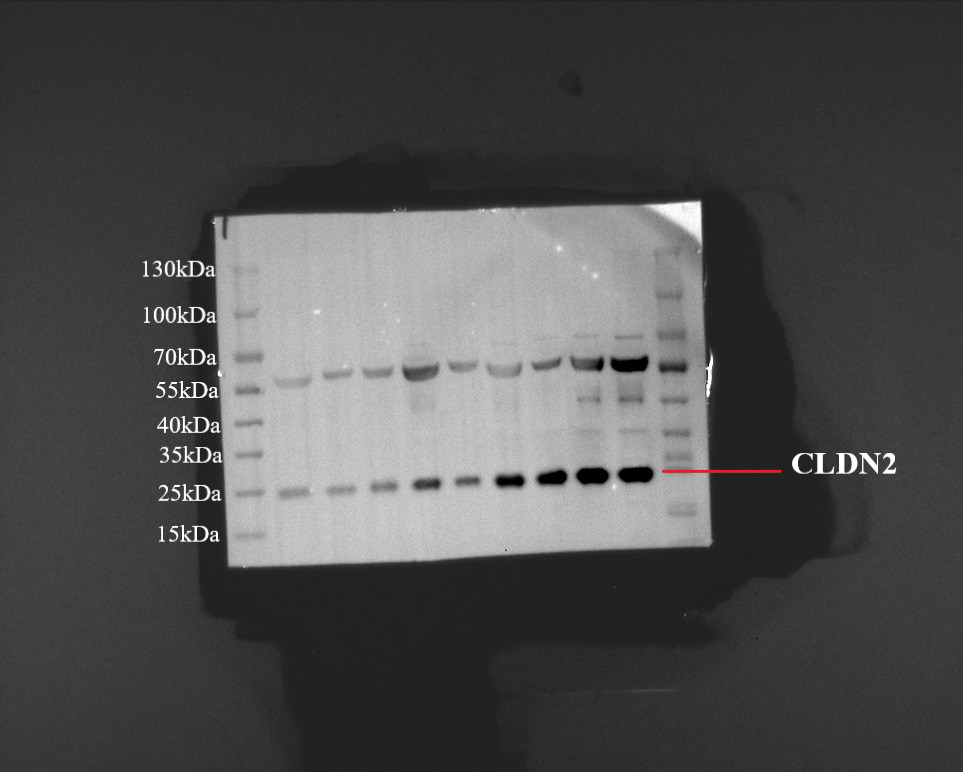

Supplement: Supplementary file 1 [file DataSheet1.ZIP › WB/CLDN2 in manuscript.tif]

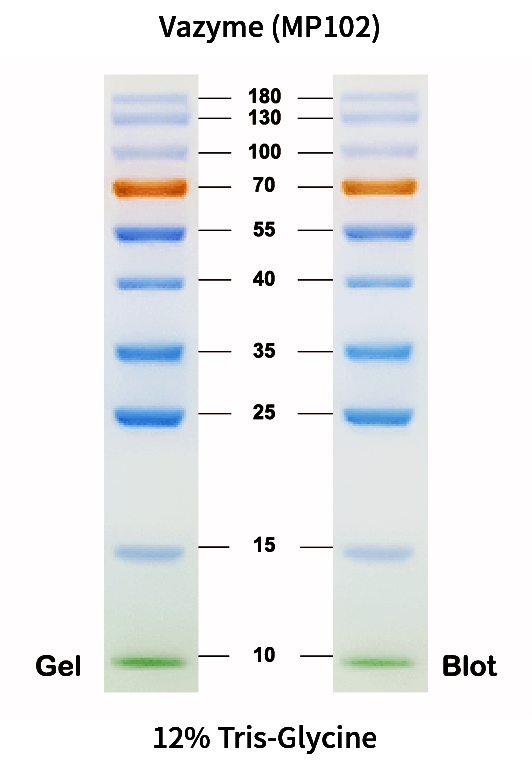

Supplement: Supplementary file 1 [file DataSheet1.ZIP › WB/Ladder.png]

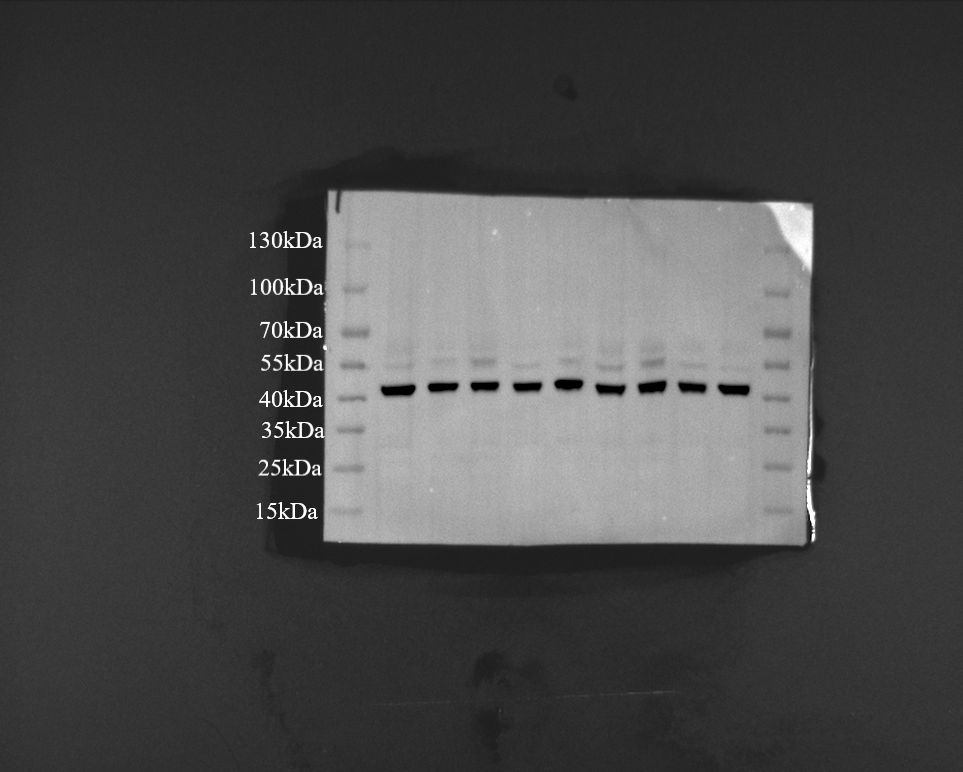

Supplement: Supplementary file 1 [file DataSheet1.ZIP › WB/a┬actin in manuscript.tif]
